# Mapping Gene Expression in Excitatory Neurons during Hippocampal Late-Phase Long-Term Potentiation

**DOI:** 10.3389/fnmol.2017.00039

**Published:** 2017-02-22

**Authors:** Patrick B. Chen, Riki Kawaguchi, Charles Blum, Jennifer M. Achiro, Giovanni Coppola, Thomas J. O'Dell, Kelsey C. Martin

**Affiliations:** ^1^Interdepartmental Program in Neurosciences, University of California, Los AngelesLos Angeles, CA, USA; ^2^Department of Psychiatry and Biobehavioral Sciences, Semel Institute for Neuroscience and Human Behavior, University of California, Los AngelesLos Angeles, CA, USA; ^3^Department of Biological Chemistry, University of California, Los AngelesLos Angeles, CA, USA; ^4^Department of Physiology, University of California, Los AngelesLos Angeles, CA, USA

**Keywords:** hippocampus, synaptic plasticity, long-term potentiation, translational regulation, gene profiling, RNA sequencing

## Abstract

The persistence of long-lasting changes in synaptic connectivity that underlie long-term memory require new RNA and protein synthesis. To elucidate the temporal pattern of gene expression that gives rise to long-lasting neuronal plasticity, we analyzed differentially-expressed (DE) RNAs in mouse hippocampal slices following induction of late phase long-term potentiation (L-LTP) specifically within pyramidal excitatory neurons using Translating Ribosome Affinity Purification RNA sequencing (TRAP-seq). We detected time-dependent changes in up- and down-regulated ribosome-associated mRNAs over 2 h following L-LTP induction, with minimal overlap of DE transcripts between time points. TRAP-seq revealed greater numbers of DE transcripts and magnitudes of LTP-induced changes than RNA-seq of all cell types in the hippocampus. Neuron-enriched transcripts had greater changes at the ribosome-loading level than the total RNA level, while RNA-seq identified many non-neuronal DE mRNAs. Our results highlight the importance of considering both time course and cell-type specificity in activity-dependent gene expression during memory formation.

## Significance statement

Hippocampal long-term potentiation (LTP) serves as one of the best physiological correlates of behavioral learning and memory, with the persistence of LTP requiring new gene transcription and translation. However, the cell types within which these gene expression changes occur and the temporal regulation of these genes following LTP induction remain poorly understood. In this study, we utilized Translating Ribosome Affinity Purification to identify global changes in the ribosome-associated RNA population within excitatory hippocampal neurons during a critical, early time window following LTP induction. Through analysis of gene expression at three times points after LTP induction, our study provides a comprehensive and quantitative portrait of the gene expression changes that occur following induction of LTP within a specific population of neurons.

## Introduction

Synaptic plasticity, the experience-dependent remodeling of neuronal connectivity, provides a means of storing memories in the brain (Milner et al., 1998). Long-term potentiation (LTP) of hippocampal synapses, an activity-dependent, long-lasting increase in synaptic strength, provides an experimental model for investigating the cellular and molecular mechanisms underlying the formation of long-term hippocampal-dependent memories (Bliss and Collingridge, 1993). The late phase of LTP (L-LTP) can be differentiated from early LTP (E-LTP) by its requirement for RNA and protein synthesis (Frey et al., 1988; Nguyen et al., 1994). Previous studies have shown that pharmacological inhibition of transcription and translation within an early time window (<~2 h) after induction of LTP inhibits the persistence of LTP, but inhibition of transcription and translation at a later time point (>~2 h) has no effect on the persistence of L-LTP (Nguyen et al., 1994; Fonseca et al., 2006; Alberini, 2008, 2009). These observations are consistent with the idea that a critical early temporal window of new transcription and translation underlies the persistence of stimulus-induced plasticity and memory (Huang et al., 1996). Many activity-dependent and LTP-induced transcripts have been identified through candidate and whole-transcriptome approaches, with demonstrated functions for some of these genes during both LTP and memory (Abraham et al., 1991; Valor and Barco, 2012; Benito and Barco, 2015). In this study, we profiled the temporal pattern of gene expression specifically within excitatory pyramidal neurons following induction of Schaffer collateral (CA3 to CA1) hippocampal LTP.

The long-lasting changes in synaptic strength that occur after a learning event require the coordinated effort of multiple cell populations. Thus, excitatory neurons, astrocytes, and inhibitory neurons have all been shown to contribute to hippocampal LTP (Ji et al., 2001; Suzuki et al., 2011). Single-cell RNA sequencing of individual neurons (Zeisel et al., 2015) and cell-type specific RNA-seq (Zhang et al., 2014) have revealed large differences in the expression of transcripts between cell types within the brain. Previous studies of hippocampal gene expression following LTP induction (Lee et al., 2005; Park et al., 2006; Coba et al., 2008; Ryan et al., 2012; Cho et al., 2015) have not, however, differentiated between changes occurring in one cell population vs. another. The presence of multiple cell types in the hippocampus diminishes the true extent of differential expression following LTP induction, as many transcripts are expressed in multiple cell types, yet may be regulated only in subsets of these cells. New methodologies have been developed in recent years for cell-type specific analysis of genome-wide changes. These include the promoter-dependent tagging of ribosomal proteins with a small protein tag (Heiman et al., 2014), which allows for downstream purification of the ribosome-associated RNA population within a genetically defined cell population from tissue composed of multiple cell types.

Here, we take advantage of Translating Ribosome Affinity-Purification Sequencing (TRAP-Seq, Heiman et al., 2008; Sanz et al., 2009) and transcriptome profiling technologies (RNA-seq) to determine the time course of differential expression following LTP induction in a cell-type specific manner. We performed TRAP-seq of the ribosome-associated population of RNA purified from excitatory neurons in hippocampal CA3/CA1 mini-slices 30, 60, and 120 min following chemical induction of LTP. We identified 899 differentially-expressed (DE) transcripts by TRAP-seq across these time points. We found that upregulated and downregulated transcripts differed in their enrichment of biological functions, and that upregulated transcripts had significantly longer untranslated regions (UTRs) than downregulated transcripts. Furthermore, we detected an enrichment of specific RNA binding protein (RBP) motifs in the 3′ UTRs of upregulated transcripts. We found that different ensembles of transcripts were DE at each time point, with further temporal profiling of DE transcripts revealing clusters of temporally-regulated transcripts that were most prominently enriched in transcription-associated genes. While there was some overlap in the DE transcripts identified at each time point by TRAP-seq (from pyramidal neurons) and RNA-seq (from all cell types), TRAP-seq detected both greater numbers of DE transcripts and greater magnitudes of differential expression at all time points. Transcripts DE exclusively in TRAP-seq were enriched for cell adhesion and cytoskeletal genes, while transcripts DE exclusively in RNA-seq were enriched for cytokine genes. Bioinformatic analyses of the RNA-seq DE transcripts identified some DE transcripts that were enriched in microglial and astrocytic cell types. Taken together, our results highlight the complexity and diversity of gene expression that occurs following LTP induction, and underscore the importance of considering both cell-type specificity and time after stimulation in determining the program of gene expression that gives rise to long-lasting brain plasticity and memory.

## Results

**Figure 1:**
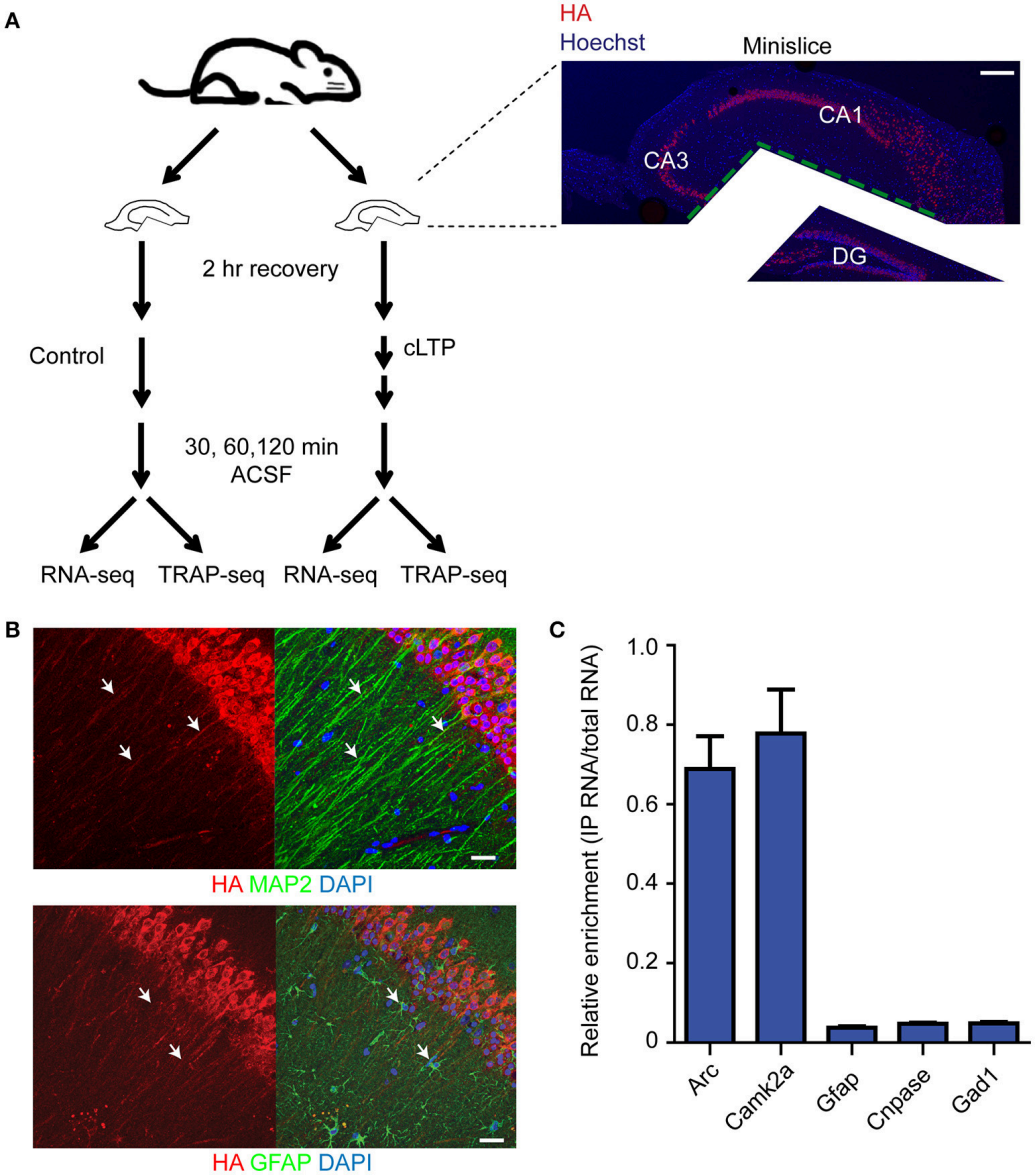
**Expression and RNA purification of ribosome-associated transcripts is specific for excitatory neurons. (A)** Diagram of the experimental design and immunohistochemistry demonstrating mini-slice preparation. Red = HA, blue = Hoechst. Scale bar = 200 μm. **(B)** HA-tagged ribosomes are expressed in excitatory neurons (labeled with Map2 antibodies with arrowheads pointing to overlap of signal, top) but not astrocytes (labeled with Gfap antibodies with arrowheads pointing to non-overlap of signal, bottom) within hippocampal mini-slices. Scale bar = 20 μm. **(C)** Immunoprecipitation and RNA purification of ribosome-associated populations from hippocampal mini-slices de-enriches for astrocytic (*Gfap*), oligodendrocytic (*Cnpase*) and inhibitory neuron-specific (*Gad1*) transcripts, but captures excitatory neuron specific transcripts (*Arc* and *Camk2a*). IP RNA values were normalized against input RNA values to generate de-enrichment ratios. *n* = 2 biological replicates, error bars = SEM.

To monitor the temporal pattern of gene expression within excitatory pyramidal neurons following induction of Schaffer collateral LTP, we prepared acute hippocampal mini-slices (which only contain the CA3/CA1 region) from adult RiboTag mice (Sanz et al., 2009) (Figure 1A). The RiboTag mouse expresses floxed HA-tagged ribosomal protein L22 (HA-L22) in cells expressing Cre recombinase, which allows for immunoprecipitation of ribosome-associated transcripts in a cell-type specific manner (Sanz et al., 2009). We crossed the RiboTag mouse line with a transgenic mouse line expressing Camk2α-cre (Tsien et al., 1996), resulting in endogenous levels of expression of HA-tagged ribosomes exclusively within excitatory pyramidal neurons of our hippocampal mini-slices (Figure 1B). We first tested the cell-type specificity of HA-L22 expression by immunoprecipitating ribosome-associated RNAs from hippocampal tissue and measuring the expression of cell-type specific transcripts by quantitative RT-PCR (qPCR). As shown in Figure 1C, the excitatory neuron-specific transcripts *Arc* and *Camk2a* were present in the affinity-purified RNA, but the inhibitory neuron-specific transcript *Gad1*, astrocyte-specific transcript *Gfap*, and oligodendrocyte-specific transcript *Cnpase* were heavily de-enriched. These results indicated that TRAP-seq from hippocampal slices from these mice would be enriched for transcripts expressed in excitatory neurons.

To optimize identification of activity-dependent changes in gene expression, we also systematically reduced the variables in our experimental protocol. We first determined the optimal recovery time following slice preparation (2 h) for induction of LTP, using qPCR to monitor injury-induced up-regulation of a set of positive control immediate early transcripts (*Arc* and *c-Fos*) and phosphorylation levels of ERK1/2 and eIF4E (Figure S1A). We next examined the effect of age on stimulus-induced gene expression, and found that the magnitude of LTP-induced upregulation of *Arc* and *c-Fos* expression was significantly greater in 2 month-old mice than in 10.5–12-month old mice (Figure S1B). While the possibility of age-related decline in activity-dependent gene regulation is of great interest for future studies, we chose to focus our efforts in this study on the LTP induced changes in ~2.5 month old mice to maximize signal to noise in RNA-seq and TRAP-seq experiments.

The use of mini-slices that only contained CA3/CA2/CA1 regions allowed us to identify changes in gene expression occurring specifically within the circuit undergoing plasticity. We tested a variety of stimulation paradigms to induce L-LTP of CA3 to CA1 synapses. We found that electrical stimulation of the CA3-CA1 synapses using 2 × 100 Hz produced significantly lower amplitude changes in *Arc* and *c-Fos* expression as measured by qPCR than did chemical induction of LTP (data not shown). We chose a cLTP induction protocol that has been shown to be transcription- and translation-dependent (modified from Chotiner et al., 2003; see Materials and Methods) and that produces LTP by triggering bursting of CA3 neurons, and thus involves synaptic mechanisms of LTP induction since removal of CA3 prevents LTP induction (Makhinson et al., 1999). Slices were prepared from 10.5 to 12 week-old RiboTag mice. Immunohistochemistry using anti-HA antibodies revealed that ~95% of pyramidal neurons within CA1 stratum pyramidale expressed HA-tagged L22 (data not shown). Slices were allowed to recover for 2 h before stimulation with the 10 min cLTP induction protocol. Perfusion with artificial cerebrospinal fluid was then resumed for 30, 60, or 120 min before the slices were snap frozen for RNA immunoprecipitation/purification, library preparation, and RNA sequencing. We isolated both ribosome-associated RNA and total RNA from each set of mini-slices, and performed TRAP-seq to monitor changes in RNA association with ribosomes specifically in excitatory pyramidal neurons and total RNA sequencing (RNA-seq) to monitor changes in the whole mini-slice transcriptome.

### TRAP-seq: LTP-induced changes in ribosome-associated transcripts in CA3 and CA1 pyramidal neurons

**Figure 2:**
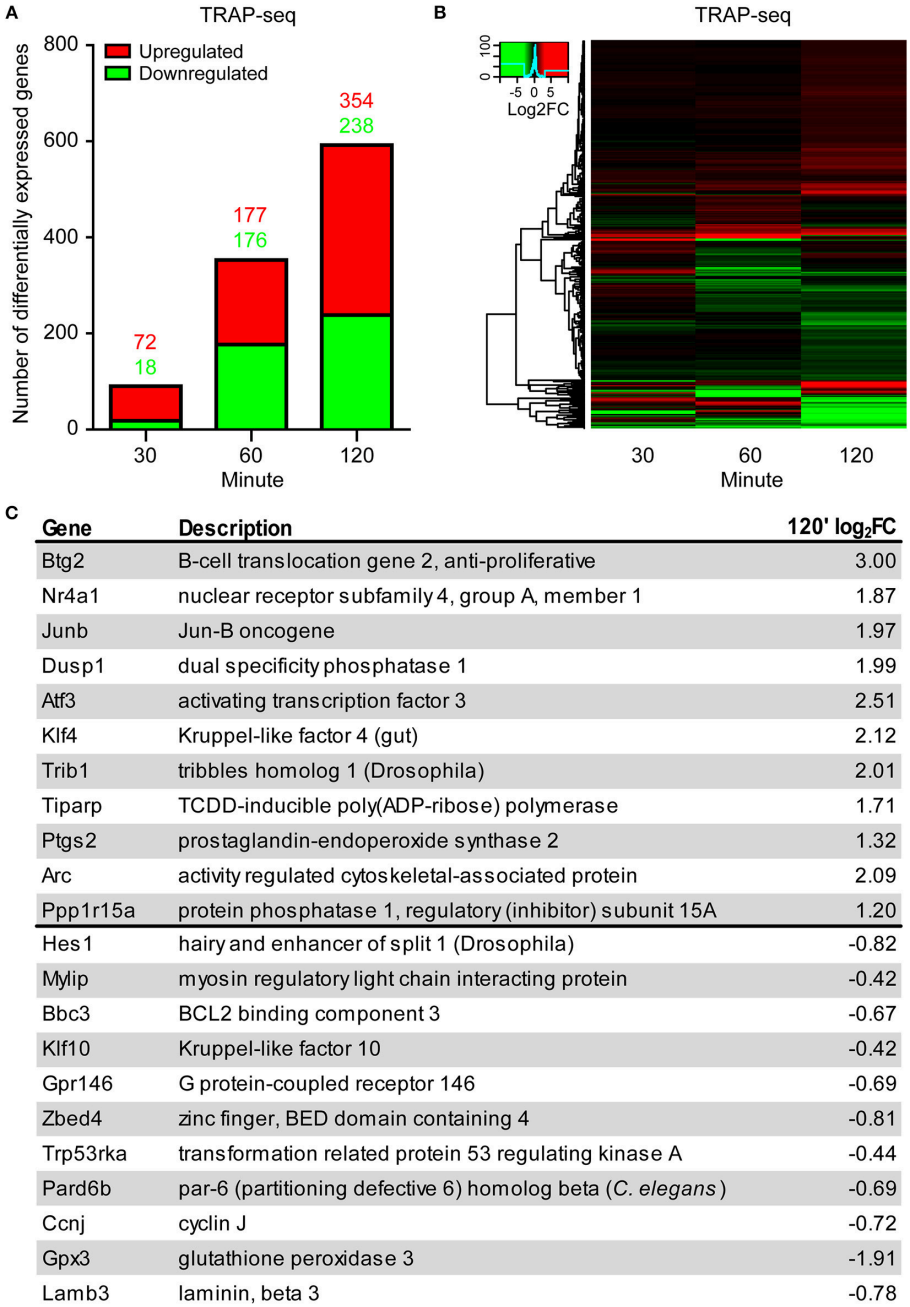
**TRAP-seq of transcripts from excitatory neurons following LTP induction at 30, 60, and 120 min reveals increasing bidirectional differential expression over time. (A)** Increasing numbers of both upregulated and downregulated DE transcripts over time. **(B)** Heat map of significant transcripts at all time points. Color key = log_2_ fold change. **(C)** List of selected significantly downregulated and upregulated transcripts at 120' post-induction with their respective log_2_ fold-changes. Transcripts selected by lowest FDR.

TRAP-seq results from the three biological replicates per time point were highly correlated (Pearson correlation coefficients of 0.99 for all time points; Figure S2). We assessed differential expression of transcripts using the Bioconductor package edgeR (Robinson et al., 2009). We considered a transcript significantly DE if the false discovery rate (FDR) was <0.1 and absolute log_2_ fold change was >0.4. We validated both differential expression and the fold-change cut-off by qPCR for 11 genes in an independently-generated biological replicate for the indicated time points (Figure S3). The number of DE transcripts identified by TRAP-seq was: 90 at 30 min, 353 at 60 min, and 592 at 120 min (Figure 2A, Table S1), indicating a gradual increase in DE transcript numbers over time following LTP induction. A large number and fraction of DE transcripts were downregulated at the two later time points (20, 50, and 40% at 30, 60, and 120 min, respectively; Figure 2A). Comparison of the magnitude of the fold-change of transcripts over time revealed that many individual transcripts underwent a time-dependent increase in amplitude of down- or upregulation (more intense green and red signals in the heat map in Figure 2B).

**Figure 3:**
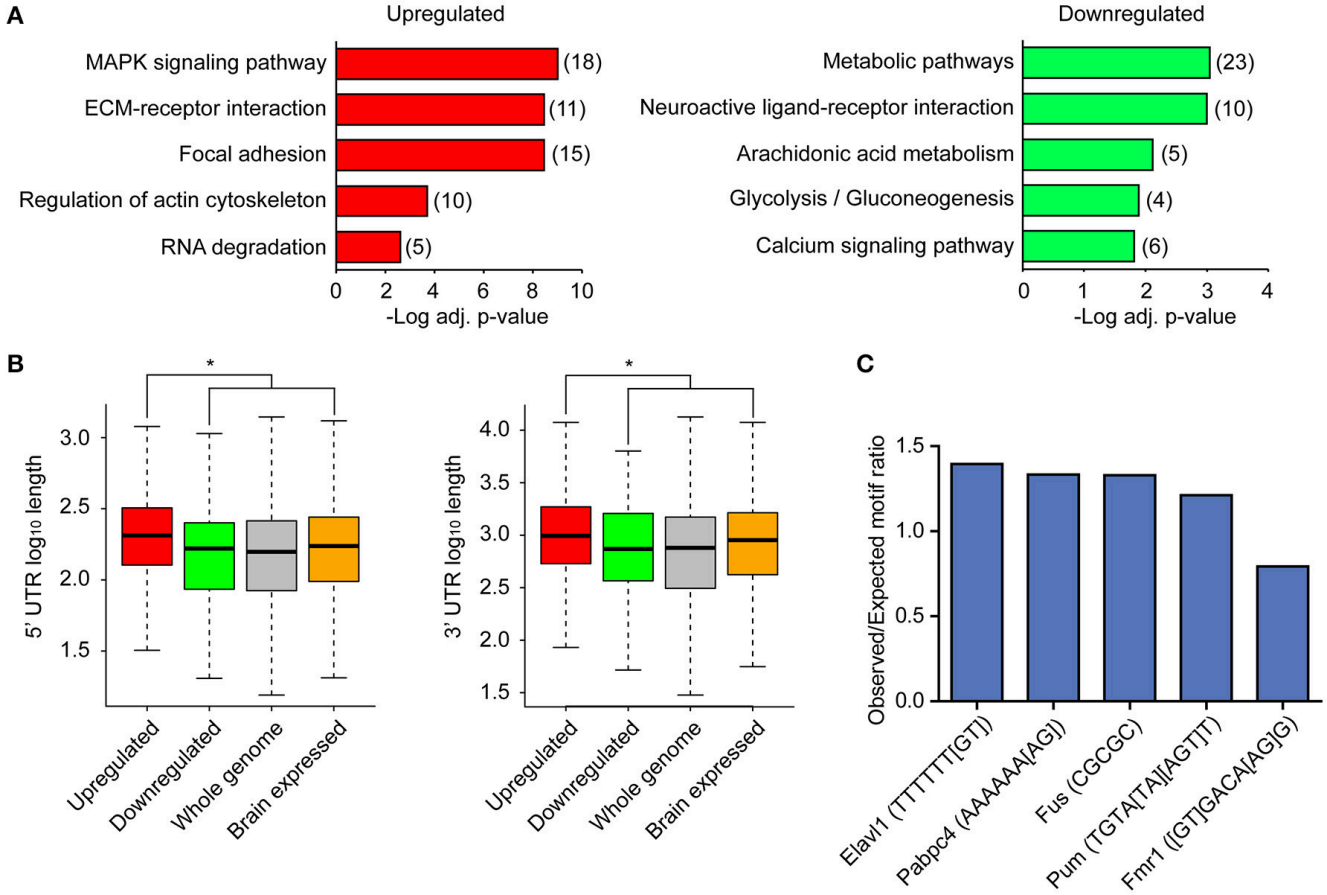
**Upregulated and downregulated transcripts differ in biological functions and in 5′ and 3′ UTR lengths, with upregulated transcripts enriched for specific RNA binding protein motifs. (A)** KEGG pathway enrichment analysis of upregulated and downregulated transcripts. The most significant pathways are shown. Red, upregulated; green, downregulated. Numbers represent number of DE transcripts per category. **(B)** Distribution of 5′ UTR lengths and 3′ UTR lengths for upregulated (red) and downregulated (green) transcripts, along with UTRs of brain-expressed transcripts and UTRs across the whole genome. Asterisk indicates significance for p < 0.01. Exact p-values for comparison between up and downregulated 5′ UTRs = 6.996e-05, 3′UTRs = 0.001748. Brain-expressed transcripts have significantly longer 3′ UTRs than the rest of the genome (Ramsköld et al., 2009). **(C)** RBP motif enrichment in 3′ UTRs of upregulated transcripts when compared to 3′ UTRs of brain-expressed transcripts. Selected RBP motifs with highest or lowest enrichment and distinct motif sequences are shown. Gene symbols were converted to mouse homologs.

To better understand the biological implications of the directionality of differential expression, we examined the upregulated and downregulated transcripts showing the most significant changes by FDR. The most significant upregulated and downregulated transcripts encoded genes involved in diverse and distinct cellular and molecular processes, with upregulated transcripts encoding transcription factors and phosphatases, and downregulated transcripts encoding G protein coupled receptors, kinases, and metabolic pathway enzymes (Figure 2C). To further explore the possibility that transcripts whose association with ribosomes was upregulated and downregulated following LTP induction are functionally distinct, we tested for enrichment of functional gene pathways using Kyoto Encyclopedia of Genes and Genomes (KEGG) pathway enrichment analysis (Table S2). We found that upregulated transcripts were more enriched for genes involved in MAP kinase signaling, extracellular matrix interactions, cell adhesion, cytoskeletal regulation and RNA degradation (Figure 3A, left), whereas downregulated transcripts were more enriched for genes involved in metabolic pathways, neuroactive ligand-receptor interactions and calcium signaling (Figure 3A, right).

We next wanted to understand whether the differential expression of these groups of ribosome-associated transcripts could be in part explained by differences within key regulatory elements of the transcripts. Direct binding of specific classes of RNA binding proteins (RBP) in the 5′ and 3′ untranslated regions (UTRs) of mRNAs has been shown to regulate translation of transcripts (Pascale et al., 2008; Lebedeva et al., 2011). Both the 5′ and 3′ UTRs of mRNAs are enriched for RBP binding sites compared to the coding region (Wilkie et al., 2003), with longer UTRs likely containing greater numbers of binding sites for RBPs. We first asked whether UTR length was correlated with down or upregulated transcripts by comparing the lengths of UTRs between transcripts that were significantly down- or upregulated. We detected significantly longer 5′ and 3′ UTRs in upregulated transcripts as compared to downregulated transcripts (Figure 3B). 5′ and 3′ UTRs were also significantly longer for upregulated transcripts as compared to previously-published brain-expressed transcript 5′ and 3′ UTRs (from Kang et al., 2011; see Materials and Methods). Since a direct interaction between RBPs, ribosomes and the 3′ UTRs of mRNAs has previously been shown to affect translation of associated transcripts following neuronal depolarization (Krichevsky and Kosik, 2001), we sought to determine whether specific RBPs play a role in the regulation of gene expression following LTP induction. To address this, we used a previously-published compendium of RBP motifs (Ray et al., 2013) to test whether there was enrichment of specific RBP motifs in the 3′ UTRs of upregulated and downregulated transcripts compared to brain-expressed transcripts. As shown in Figure 3C and Table S3, we found that U-rich motifs were significantly overrepresented in the 3′ UTRs of upregulated transcripts, as were the binding motifs for several RBPs known to regulate RNA metabolism in neurons, including CPEB2 and 4 and Pum (Darnell and Richter, 2012), Hu/Elavl proteins (Lee et al., 2015), and Fus (Ling et al., 2013). Binding motifs for only two RNA binding proteins, Vts1p (yeast, mammalian Smaug or Samd4) and Fmr1 were underrepresented in the 3′ UTRs of upregulated transcripts. A smaller number of significantly overrepresented motifs were present in downregulated transcripts (Table S3). We also analyzed RNA binding motifs in the 5′ UTRs, and identified 38 overrepresented RBP binding sites, including Fus, CPEB4, and Hu/Elav1 proteins and 11 underrepresented RBP binding sites. A smaller number of overrepresented and underrepresented RBP binding sites were detected in the 5′ UTRs of downregulated transcripts. Many of the RBPs in Table S3 were expressed within excitatory neurons from our TRAP-seq data, suggesting that RBP binding may serve as one mechanism of regulating transcript expression following LTP induction.

**Figure 4:**
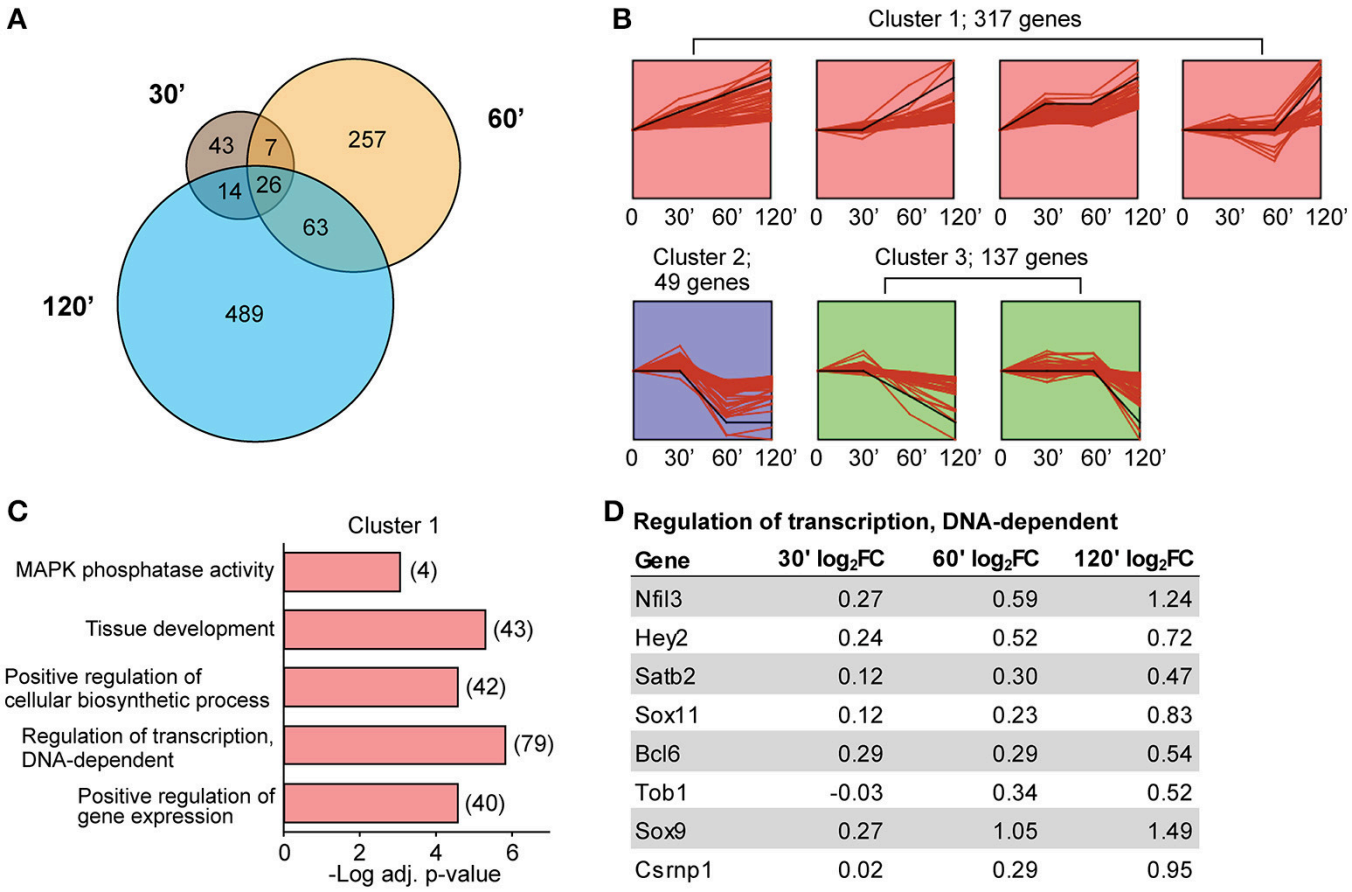
**Minimal overlap of DE transcripts between time points and enrichment of transcription-associated genes within temporally co-regulated transcripts. (A)** Numbers of significant transcripts at each time point and shared transcripts between time points. **(B)** STEM analysis grouping of significant differentially-expressed transcripts into three temporal profile clusters. Black line represents model temporal profile. **(C)** Selected GO terms enriched in cluster 1 transcripts based on significance, gene numbers, specificity of GO term, and biological interest. Numbers represent number of DE transcripts per category. Clusters 2 and 3 did not show enrichment for any GO terms when taken separately or combined. **(D)** The log_2_ fold-changes at each time point for genes belonging to the GO category “Regulation of transcription, DNA-dependent.”

Our finding that the number of DE transcripts increased over time (Figure 2A) led us to more carefully examine the temporal characteristics of differential expression following LTP induction. Although the general direction of change was consistent across time for most transcripts (Figure 2B), there was only marginal overlap of significant DE transcripts between time points (Figure 4A). Despite significant overlap of DE transcripts between time points (*p* < 1.0^−30^ for paired time point overlap), the proportion of exclusively DE transcripts at their respective time points was 48% of DE transcripts at 30 min, 73% of DE at 60 min, and 83% of DE transcripts at 120 min. To more rigorously characterize the temporal dynamics of differential expression following LTP induction, we utilized Short Term Expression Miner (STEM, Ernst and Bar-Joseph, 2006) to group DE transcripts based on the similarity of their temporal expression patterns. Previous studies have shown that temporally co-regulated transcripts encode genes with related biological functions (Tornow and Mewes, 2003; Di Giovanni et al., 2004); this approach would therefore increase the likelihood of detecting biologically significant processes related to LTP. STEM analysis revealed that most transcripts clustered into a temporal profile representing a general upregulation over time (cluster 1, Figure 4B, Table S4). The other two clusters contained transcripts that were largely downregulated over time (clusters 2 and 3, Figure 4B, Table S4). We next tested for functional enrichment within the groups of transcripts associated with each cluster. To further increase the specificity of this analysis for processes related to LTP, we utilized a background gene list derived from our TRAP-seq data that included transcripts with a minimal expression level of an average of 20 normalized reads across all samples. Gene ontology (GO) analysis revealed that cluster 1 was heavily enriched for processes involved in transcription and transcriptional regulation (Figure 4C, Table S5), while GO analysis of clusters 2 and 3 revealed no significant enrichment. Genes within a selected enriched transcriptional regulation GO category (Figure 4D) demonstrated a clear increase in log_2_ fold change over time; only a few of these transcription factors have been previously implicated in plasticity. Importantly, some of these genes fell below our significance criteria at individual time points but are included within significant STEM profiles, confirming that analysis of DE at multiple time points is more sensitive than single time point experiments in identifying co-regulated transcripts sharing biological functions.

**Figure 5:**
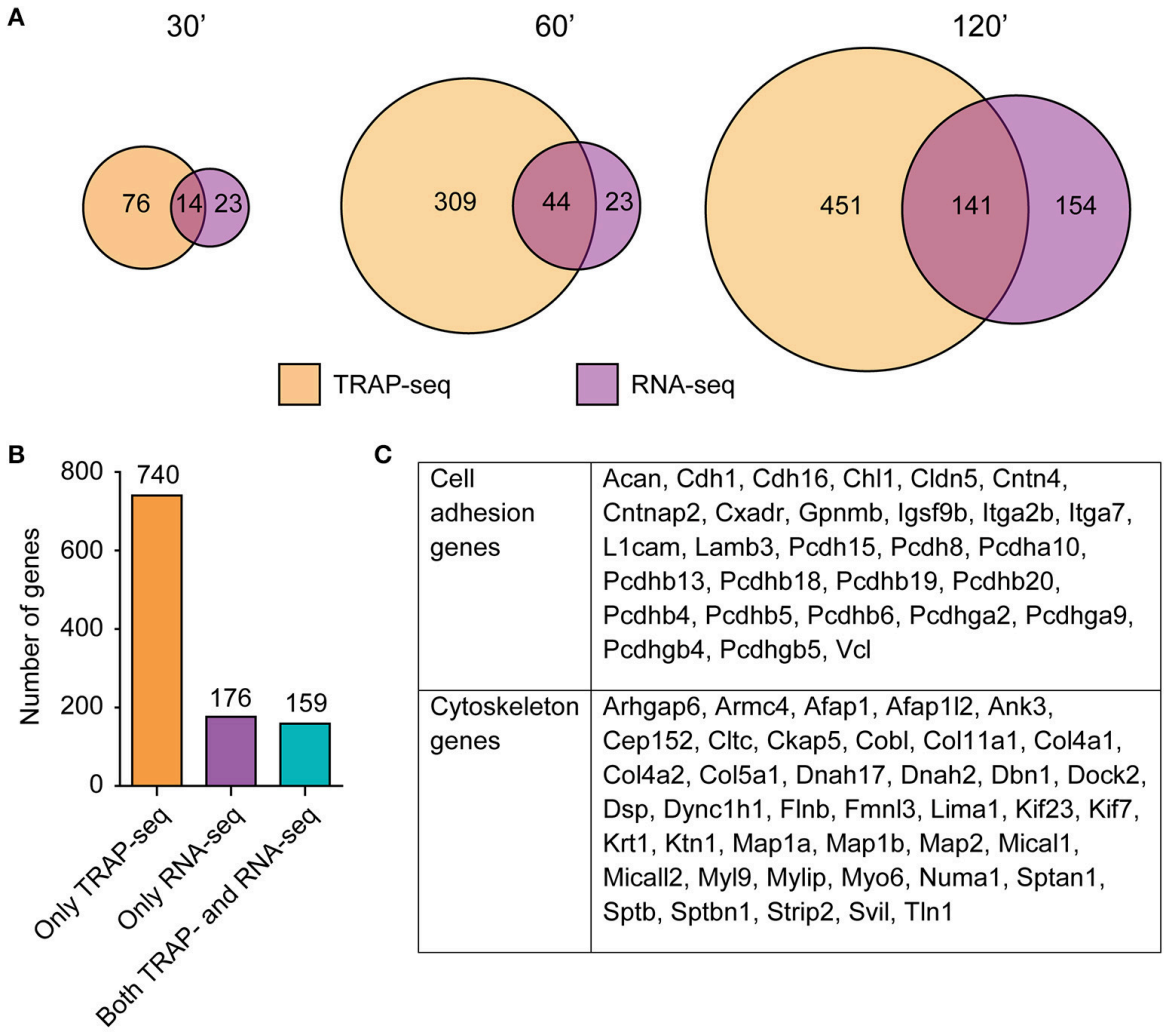
**Marginal overlap of DE transcripts between TRAP-seq and RNA-seq at each time point, with a specific enrichment of cytoskeletal and cell adhesion genes identified in transcripts only DE in the ribosome-associated population. (A)** Overlap of significant transcripts at each time point between RNA populations. **(B)** Numbers of transcripts that were identified in one or both populations, collapsed across time. **(C)** Enriched functional categories within exclusively ribosome-associated DE transcripts, with genes corresponding to each category. Genes were assigned based on PANTHER annotation in conjunction with WebGestalt results.

In addition to TRAP-seq, we also purified and sequenced total RNA (composed of RNA from all cell types) from the same set of mini-slice homogenates used to generate RNA for TRAP-seq, allowing a direct comparison between the two techniques from a single biological sample (Table S6). Overall, more DE transcripts were identified by TRAP-seq compared to RNA-seq at every time point following LTP induction (Figure 5A), though the time-dependent increase in DE transcript numbers and magnitude of differential expression we observed by TRAP-seq was also observed by RNA-seq (Figure 5A, Figure S4). There was significant overlap of DE transcripts in TRAP-seq vs. RNA-seq at each time point (*p* < 1.0^−20^ for overlap at each time point); however, the overlap was modest, with a smaller fraction of TRAP-seq DE transcripts being detected by RNA-seq at all time points. Another difference we observed was that unlike TRAP-seq, a majority of DE transcripts detected by RNA-seq at earlier time points was also DE at later time points (Figure S4, Figure 4A). Differences in fold-change between RNA-seq and TRAP-seq, and between time points, were validated by qPCR of a separate biological replicate for both larger and smaller fold-change magnitudes close to our fold-change cutoff (Figure S3).

When collapsed across all time points, a total of 740 transcripts were DE only by TRAP-seq, while 176 transcripts were DE only by RNA-seq and 159 were DE in both (Figure 5B, Table S7). This indicated that only a subset of TRAP-seq and RNA-seq DE transcripts were shared, and that each technique identified different sets of transcripts. Interestingly, the three categories of genes—DE in both TRAP-seq and RNA-seq, DE in only TRAP-seq, or DE in only RNA-seq—were enriched in distinct biological functions. DE transcripts in both TRAP-seq and RNA-seq were enriched for transcription associated genes (Figure S5), DE transcripts in only RNA-seq were enriched for cytokine signaling pathways (Figure S5), while DE transcripts in only TRAP-seq were enriched for cell adhesion and cytoskeletal genes (Figure 5C). Of note, members of the cadherin and protocadherin families as well as members of several different classes of microtubule-binding and motor proteins (e.g., MAP2 and MAP1a/1b, Myo6 and Dnah2) were DE only within the TRAP-seq data. These families of genes are involved in biological processes important in LTP (Correia et al., 2008; Tai et al., 2008), and certain DE transcripts in these enriched categories have been shown to be critical for excitatory transmission and LTP (Yamagata et al., 1999; Osterweil et al., 2005).

**Figure 6:**
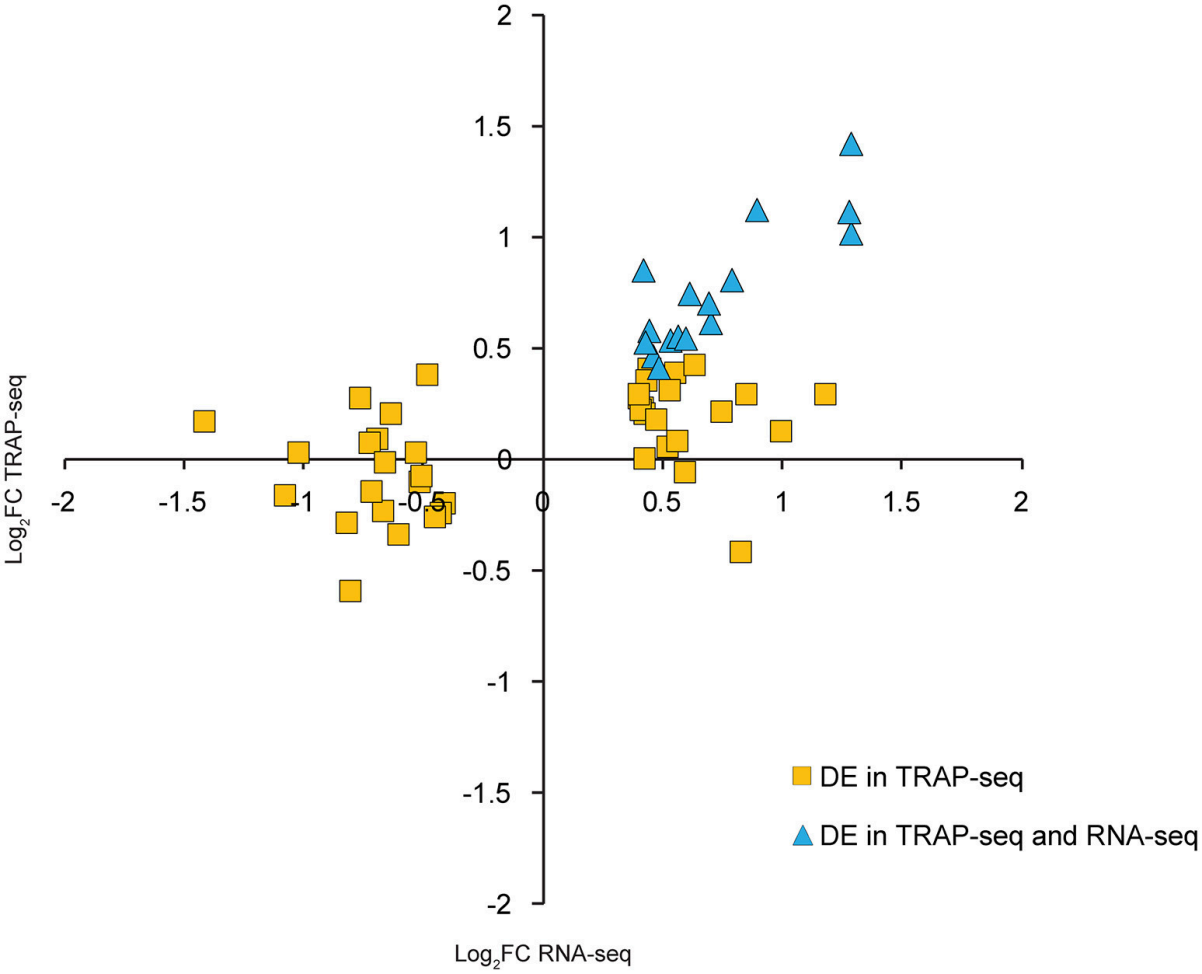
**Neuron-enriched transcripts have significantly greater fold-change magnitudes by TRAP-seq than RNA-seq following LTP induction**. Each point represents one transcript that was both neuron-enriched and DE by TRAP-seq.

Interestingly, transcripts known to be neuron-specific, such as *Map2* and *Map1b*, were DE by TRAP-seq but virtually unchanged by RNA-seq. This raised the possibility that transcripts could be regulated differently at ribosome-associated and total RNA steady states; specifically, changes in the ribosome-associated population would not necessarily require corresponding changes in the total RNA concentration. To address this possibility using a more unbiased approach, we used a bioinformatics approach to identify neuron-enriched transcripts by looking for transcripts whose basal expression was overrepresented by TRAP-seq as compared to RNA-seq (see Materials and Methods for details). We confirmed the neuron-enriched expression of these transcripts using the Allen Brain Atlas (http://mouse.brain-map.org/); of the 30 DE neuron-enriched transcripts that had detectable expression by the Allen Brain Atlas, only 1 was excluded for being non neuron-enriched. We then compared differential expression of this set of transcripts in the TRAP-seq and RNA-seq data. From this, we identified 16 transcripts that were DE at comparable levels by both RNA-seq and TRAP-seq (mainly comprised of immediate-early genes that are rapidly transcriptionally/translationally upregulated; Abraham et al., 1991) and 43 transcripts that were only DE by TRAP-seq (Table S8). Taken together, the magnitude of differential expression of these transcripts by TRAP-seq was significantly greater than RNA-seq, (Figure 6, *p* < 0.0001; Wilcoxon signed rank test). This greater magnitude of differential expression was observed for both upregulated and downregulated transcripts.

**Figure 7:**
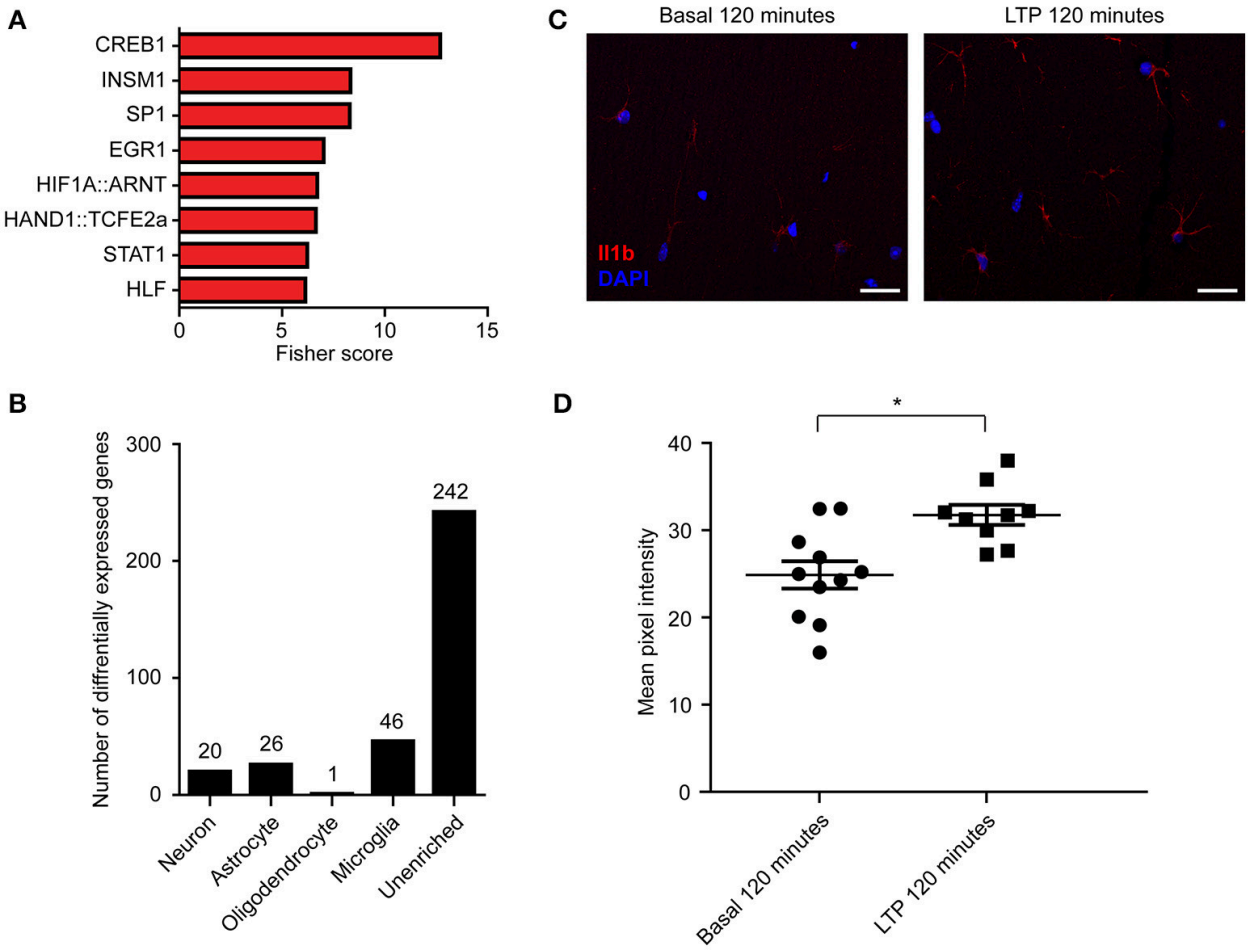
**Enrichment of transcription factor binding in RNA-seq DE transcripts suggests potential regulators of differential expression, and some DE transcripts are enriched in non-neuronal cell types. (A)** Transcription factor enrichment ordered by Fisher score, which represents overrepresentation of transcripts containing motif over background levels. TRANSFAC analysis identifying motif overrepresentation within our significant transcripts was also performed (Table S9B). **(B)** Numbers of DE transcripts that were enriched in each cell type (see Section Materials and Methods). **(C)** Representative immunohistochemistry for the microglial-enriched transcript Il1b demonstrating an increase in Il1b protein level (red) following LTP induction at 120′. **(D)** Grouped data from Il1b immunostaining (*n* = 2 animals, 11 slices). Scale bar = 20 μm. Data points represent the mean pixel intensities from ROIs around cell bodies and processes from single slices. Asterisk indicates *p* < 0.05; exact *p* = 0.0122. Error bars = SEM.

Given that specific transcription factors have been shown to coordinate the transcription of multiple important transcripts for LTP and memory (Alberini, 2009), we next sought to determine whether DE transcripts identified through RNA-seq were coordinately regulated by distinct transcription factors. Using two separate bioinformatic approaches to assess enrichment of known targets or binding motif sequence enrichment—oPOSSUM-3 (Kwon et al., 2012) (Figure 7A, Table S9A) and TRANSFAC (Matys et al., 2006) (Table S9B)—we identified CREB1 as the transcription factor with the most enriched binding sites, consistent with previous literature on the critical role of CREB1-mediated transcription following LTP induction (Deisseroth et al., 1996). Other transcription factors with highly enriched targets within our DE transcripts included STAT1 and EGR1, which have previously been implicated in plasticity (Bozon et al., 2002; Barbosa et al., 2008), and NFAT1 and MZF1 (Tables S9A,B), transcription factors that have not been reported to be involved in neuronal plasticity but that are expressed in hippocampal tissue as determined by our RNA-seq data.

To our surprise, the GO categories of DE transcripts identified only through RNA-seq included processes not generally associated with neurons (Figure S5). Specifically, we detected an enrichment of biological processes involved in immune functions, such as those involved in cytokine signaling pathways. This raised the possibility that the induction of LTP triggered DE of transcripts not only in neurons, but also in other cell types. To explore this possibility, we utilized published transcriptional profiles from individual cell types in the mouse brain (Zhang et al., 2014) to generate a list of transcripts that were enriched in neurons, astrocytes, myelinating oligodendrocytes, or microglia (see methods). We then determined the number of DE transcripts that were enriched in each cell type (Figure 7B, Table S10). While most DE genes could not be attributed to a specific cell type, microglia had the highest number of cell-enriched DE transcripts (46 transcripts), including chemokine ligands and receptors. To further validate the cell-type specificity of gene expression following LTP induction, we stained for the significantly DE microglial protein Il1b by immunohistochemistry at 120 min post-LTP induction and detected significantly increased immunoreactivity of Il1b protein in microglia following LTP induction (Figures 7C,D). These results suggest that a portion of the observed changes in total RNA were due to LTP-induced changes in the transcriptome of specific non-neuronal cell types. Taken together, these results underscore the need for cell-type specific studies of gene expression to understand the changes in gene expression that give rise to persistent forms of learning-related plasticity.

## Discussion

Decades of research have aimed to identify the specific activity-dependent genes whose expression gives rise to long-term plasticity and long-term memory. Many activity-dependent immediate early genes such as *Arc, c-Fos*, and *Egr1*, were initially identified using subtraction-cloning approaches comparing stimulated vs. unstimulated hippocampi (Nedivi et al., 1993). The development of microarray and next-generation sequencing technologies has permitted systematic and quantitative genome-wide analysis of the changes in gene expression that occur at various time points following the induction of LTP (Park et al., 2006; Coba et al., 2008; Ryan et al., 2012; Cho et al., 2015). Our intent in this study was to use sensitive, genome-wide approaches to optimize the discovery of activity-dependent genes and to thereby provide a comprehensive picture of the changes in gene expression that occur in excitatory neurons following induction of plasticity. We utilized a cLTP induction protocol to ensure that the maximal number of synapses would be potentiated for downstream gene expression analysis, and used CA3/CA1 mini-slices to focus our transcriptome analyses specifically on the pathway undergoing plasticity. Cell-type specific TRAP-seq was used to focus on gene regulation specifically within excitatory neurons. Although analysis of gene expression following learning in the behaving animal might be considered more relevant to identifying the genes whose expression lead to long-term memory formation, the sparse encoding of memory within neural circuits (Chawla et al., 2005; Rumpel et al., 2005) reduces signal to noise and thus impedes the identification of activity-dependent alterations in gene expression. For example, we identified a total of 335 DE transcripts by RNA-seq across all time points (Table S6), but a recent study utilizing RNA-seq to assay gene expression in hippocampus following fear conditioning (Cho et al., 2015) only identified 112 DE transcripts with more time points included and even less stringent criteria for differential expression than our study. In another recent publication using TRAP-seq to identify changes in ribosome-association of mRNAs in pyramidal cell dendrites following contextual fear conditioning, the variation between the biological replicates within the control and fear-conditioned animals was as large as the variation between the control and fear-conditioned animals (Ainsley et al., 2014; also E. M. Schuman comment in PubMed Comments). We believe that using TRAP-seq to systematically detect DE genes following LTP induction in a reduced slice preparation provides a fruitful first step in identifying specific genes that can subsequently be studied during learning and memory in the animal.

### Temporal regulation of gene expression following LTP induction

Studies using transcriptional and translational inhibitors have given rise to the idea that changes in gene expression only during the first 2 h following stimulation are critical to the persistence of hippocampal LTP (Frey et al., 1988; Nguyen et al., 1994; Nguyen and Kandel, 1997; Fonseca et al., 2006). The results of our TRAP-seq and RNA-seq experiments call this idea into question. We observe a time-dependent increases in the number of DE transcripts, with the greatest number of DE transcripts detected at 120 min, a time point when LTP persistence has been reported to lose sensitivity to translational and transcriptional inhibitors. Our results add to a growing list of studies (Bourtchouladze et al., 1998; Taubenfeld et al., 2001; Bekinschtein et al., 2007; Katche et al., 2013; Bambah-Mukku et al., 2014) that challenge the idea of a relatively short and early time window in which gene expression is exclusively occurring. Transcripts DE only at 120 min could serve as novel markers for late-phase LTP, as current proxies for synaptic plasticity such as *c-Fos* and *Arc* are transcribed immediately following non-LTP inducing levels of activity, and are not always associated with long-lasting plasticity and memory (unpublished data, Kim et al., 2010). Further studies examining the temporal patterns of gene expression during even later time points may provide greater insights into the temporal dependence of gene expression following LTP and learning.

One unexpected aspect of temporal regulation that emerged from our TRAP-seq data was that a relatively small (but significant) fraction of transcripts was DE across multiple time points (Figure 4A). This finding indicates that regulated ribosome association of mRNAs at later time points is not simply a continuation of regulated ribosome association at earlier time points, but rather that there are discrete rounds of differential ribosome association of transcripts following LTP induction. In contrast, RNA-seq showed a much greater proportion of temporal overlap between DE transcripts than TRAP-seq (Figure 4A, Figure S4), consistent with a more continuous pattern of transcriptional than translational regulation following LTP stimulation. This may be expected given the more dynamic kinetics of ribosome-association compared to total RNA regulation.

### Cell-type specificity of gene regulation underlying LTP

In this study, we measured differential expression occurring specifically within excitatory neurons following LTP induction using TRAP-seq, which revealed a large number of upregulated and downregulated DE transcripts that were not detected by RNA-seq from all cell types (Figures 2, 5A). This difference can in part be explained by differences in cell-type composition, with RNA-seq detecting fewer DE transcripts due to a dilution effect from other cell types. For example, transcripts such as *c-Fos* are DE in neurons but are also highly expressed within astrocytes (Arenander et al., 1989; unpublished data), which would diminish the detectability of DE by RNA-seq relative to excitatory neuron-specific TRAP-seq. It is unclear whether DE of transcripts expressed within neurons also occurs within other cell types. Interestingly, our bioinformatic identification of cell-type enriched transcripts that were DE by RNA-seq suggests that differential expression occurs in non-neuronal cell types following LTP induction, though most DE transcripts could not be ascribed to a single cell type using this approach. We detected 46 microglia and 26 astrocyte-enriched transcripts that were differentially expressed in our RNA-seq experiments, and demonstrated a corresponding microglial protein level expression change for the microglial-enriched transcript *Il1b* (Figures 7B–D). Astrocytic functions have been shown to be important for LTP and memory (Suzuki et al., 2011) and microglia have been shown to regulate activity-dependent synaptic pruning (Schafer et al., 2012). In this context, our findings highlight the importance of analyzing gene expression in non-neuronal cell types during forms of synaptic plasticity such as hippocampal LTP.

### Gene expression regulation following LTP induction

Gene expression is a coordinated process involving multiple layers of regulation, including transcriptional and post-transcriptional, with changes in total RNA following LTP induction traditionally thought to be required for changes in protein level. RNA-seq measures the concentration of total RNA, which reflects transcriptional regulation as well as RNA stability, while TRAP-seq measures the concentration of ribosome-associated mRNAs, which reflects not only mRNA concentration but also post-transcriptional regulation at the level of ribosome association. The difference in 5′ and 3′ UTR lengths between upregulated and downregulated transcripts of the ribosome-associated mRNAs, and enrichment of specific RBP motifs within their 3′ UTR sequences (Figures 3B,C), provide clues into the types of post-transcriptional regulation mechanisms that may underlie the upregulation and downregulation of these transcripts. Further analysis taking into consideration RNA secondary structural motifs, which are known to play critical roles in regulatory mechanisms such as RBP binding (Ricci et al., 2013), will be important for identifying other mechanisms of post-transcriptional regulation following LTP induction.

We note that the fold-change magnitude of most DE transcripts was modest, with most transcripts not changing more than an absolute log_2_FC of 1. While transcripts undergoing larger fold-change differential expression are better targets for some types of downstream experimental analyses, and are easier to confirm using less quantitative methodologies, such as immunoblotting or immunohistochemistry, DE transcripts with smaller fold-changes may still play a critical role in LTP. Smaller fold-changes may in fact be expected for the fine-tuning of biological systems, such as during synaptic plasticity, rather than a complete overhaul, such as during developmental changes in cell fate. Importantly, despite the low magnitude of fold change, differential expression was highly reproducible, not only between biological replicates used for RNA-seq and TRAP-seq, but also in the biological replicates used for qPCR confirmation (Figure S3).

Local protein synthesis has also been shown to be critical for LTP, which can occur in a transcription-independent manner (Kang and Schuman, 1996). A number of the DE transcripts that were identified only by TRAP-seq have also previously been shown to be localized to dendrites (Cajigas et al., 2012, Table S11), raising the possibility that their differential expression occurs at the ribosome-associated level in dendrites as well as in cell somata. Thus, the magnitude of differential expression we detected in many of these DE transcripts may in fact be changing at greater levels specifically within subcellular compartments; this has been demonstrated with translation of Camk2α protein only occurring locally within dendrites following stimulation (Ouyang et al., 1999). We also note that TRAP-seq may also incorporate measurements of ribosomal stalling—a mechanism used by FMRP to decrease rates of translation (Darnell et al., 2011)—which would affect the correlation of TRAP-seq changes and protein level changes for some of these transcripts. All of these issues highlight the necessity of better tools to dissect the different layers of regulation during LTP. For example, combining TRAP-seq with ribosome profiling provides a means of differentiation between ribosome stalling and procession, while methods like BONCAT allow for cell-type specific proteomic measurements of activity-dependent changes in protein expression (Müller et al., 2015).

When comparing the differential expression magnitude of neuron-enriched genes, we found that DE transcripts had significantly larger fold-change magnitudes at the ribosome-associated level than the total RNA level. This may result from (1) the faster kinetics of ribosome-associated regulation compared to regulation of total RNA steady states; (2) the lower basal copy number of ribosome-associated transcript compared to total RNA copies; and/or (3) distinct stimulus-induced post-transcriptional (and transcription-independent) regulation. Indeed, the fact that we detected such a large number of downregulated transcripts at all time points by TRAP-seq but not by RNA-seq (Figure 2A, Figure S4) is consistent with the faster kinetics of ribosome association (as compared to regulation of total RNA levels). These results suggest that neuronal stimulation can regulate transcript translation independently of changes in transcript concentration, and focuses attention on activity-dependent translational regulation. This is consistent with studies of stimulus-induced gene expression in a homogenous cell population that revealed significantly greater changes in polysome-associated RNA than total RNA (Tebaldi et al., 2012). Although we were very conservative in our criteria of neuron-enrichment, we cannot fully rule out the possibility that there may be expression of these transcripts in non-neuronal cells. To systematically test the extent of differential regulation at the total RNA and ribosome-loaded level, cell-type specific total RNA analysis is needed for a direct global comparison of the kinetics between the two RNA populations (Gay et al., 2013). This type of analysis will likely require the development of new technologies that reduce the amount of starting material required for cell-type specific RNA-seq. Thus, performing TRAP-seq without amplification required 20 hippocampal slices per condition per replicate (40 per experiment per replicate), and adding cell-type specific RNA sequencing using methods such as 4tU labeling (which would label only a fraction of the total RNA), would require an even greater number of animals per replicate, greatly reducing the feasibility of these experiments.

## Concluding remarks

The design and scope of this study was not to hand-select candidates and assay their function in LTP. Previous studies utilizing this approach have proven invaluable in implicating different molecules important for LTP, but a unified model of the molecular requirements for LTP is lacking (Sanes and Lichtman, 1999; Lisman et al., 2003). The large degree of time-dependent differential expression of hundreds of transcripts encoding genes involved in diverse functions raises questions about the interpretation of single-gene studies in understanding mechanisms of LTP and memory. The results presented here paint a clear picture of the sprawling complexity of gene regulation, even within only a single cell type, following LTP induction. When studying a single gene within this large web of interconnected genetic pathways, manipulations of a single gene may have widespread consequences—even altering the entire molecular network that is involved in plasticity. Thus, experimentation with individual candidates should involve more rigorous follow-up profiling of the downstream consequences on gene expression within the cell. Our results would argue that detailed, naturalistic studies are necessary to first provide an important molecular blueprint by which candidate-based studies can be interpreted.

## Materials and methods

### Generation of RiboTag × Camk2α-Cre mice

Both RiboTag and T29/Camk2α-Cre mice were purchased from Jackson Laboratories (RRID:IMSR_JAX:011029, RRID:IMSR_JAX:005359). Both male and female 8–12 week old Ribotag × Camk2α-Cre double heterozygotes were used to prepare hippocampal mini-slices.

### CA3/CA1 mini-slice preparation and cLTP induction protocol

400 μm thick hippocampal CA3/CA1 mini-slices were prepared from hippocampi of RT x Camk2α-cre double heterozygotes. The dentate gyrus was microdissected following slicing and mini-slices were allowed to recover for 2 h at 30°C in interface-type chambers with oxygenated (95% O_2_/5% CO_2_) ACSF containing 124 mM NaCl, 4 mM KCl, 25 mM NaHCO_3_, 1 mM NaH_2_PO_4_, 2 mM CaCl_2_, 1.2 mM MgSO_4_, and 10 mM glucose. LTP was induced by submerging slices in 50 μM forskolin (in normal ACSF containing 0.2% DMSO from addition of forskolin) for 5 min, followed by 30 mM KCl/10 mM Ca2+/50 μM forskolin in 0 Mg2+ ACSF for 5 min. Control solution contained 0.2% DMSO in normal ACSF for 10 min. Slices were perfused with normal ACSF after LTP induction, with time 0 starting immediately after applying the K^+^/Ca^2+^/forskolin solution. Slices were snap frozen in crushed dry ice at the specified time points, and stored at −80°C until RNA purification.

### Immunoprecipitation (IP) and RNA purification of ribosome-loaded/total RNA populations

Frozen slices were homogenized using a pestle in IP buffer which contained 50 mM Tris-HCl pH 7.4, 100 mM KCl, 12 mM MgCl_2_, 1% NP-40, 1 mM DTT, 200 U/mL Protector RNAse inhibitor (Roche), 1 mg/mL heparin, 100 μg/mL cycloheximide, cOmplete protease inhibitor tablet (Roche). Pre-conjugated HA beads (EZview Red Anti-HA Affinity Gel, Sigma, RRID:AB_10109562) were washed twice with IP buffer before use. After homogenization, the homogenate was centrifuged twice at 10,000× g at 4°C. 1/10 of the homogenate at this step was immediately placed into Trizol (Invitrogen) for total RNA extraction. The remaining homogenate was incubated with washed HA beads overnight at 4°C. The following day the beads were washed 3× in a high salt buffer composed of 50 mM Tris, 300 mM KCl, 12 mM MgCl_2_, 1% NP-40, 1 mM DTT, and 100 μg/mL cycloheximide. RNA was eluted from the beads using 350 μL Buffer RLT + BME supplementation from the RNeasy Micro kit (Qiagen). Eluted IP RNA was column purified according to the kit protocol. Total RNA was purified through a combined Trizol and RNeasy kit protocol: after the spin gradient step in the Trizol purification, the aqueous layer was taken and mixed 1:1 with 100% EtOH and purified on a spin column. For each biological replicate, four mice were used, with ~20 slices per treatment condition. TRAP purification yielded ~200 ng RNA per 20 mini-slices, and total RNA from 1/10 of this homogenate yielded ~400 ng RNA. RNA quantification was done with the Qubit RNA HS assay (Thermo Scientific).

### Immunohistochemistry of RiboTag × Camk2α-Cre mice

Hippocampal slices were fixed in 4% PFA for 2 h at room temperature, rinsed 2× with PBS, covered in HistoGel (Thermo Scientific), then paraffin-embedded. 4 μm thick paraffin sections were deparaffinized and underwent heat-induced antigen retrieval. Sections were permeabilized with 0.1% TX-100 at room temperature for 30 min, then blocked in 10% goat serum at room temperature for 60 min. Slices were incubated with mouse anti-HA antibody (Covance, 1:1000, RRID:AB_291263) and rabbit anti-MAP2 (Millipore, 1:2000, RRID:AB_91939) overnight at 4°C, and with secondary antibodies (1:2,000) at 2 h at room temperature and counterstained with Hoechst (1:1,000).

Rabbit anti-Il1b (Abcam, 1:200, RRID:AB_308765) was used to confirm a microglial DE transcript. Images were taken from control- and LTP-treated slices from two animals at the 120 min time point, with 3–4 slices per animal per condition. ROIs were drawn around staining from cell bodies and processes, and the mean pixel intensity was taken for each ROI using ImageJ software. Each data point represents one slice. Mann-Whitney nonparametric test was used to determine significance.

### Quantitative PCR for non-specific transcript depletion, cLTP induction, validation

To assay depletion of non-specific transcripts, 50 ng of total RNA and TRAP RNA were reverse transcribed into cDNA using SuperScript III with random hexamer primers (Invitrogen). Technical triplicate reactions for each primer set (*Gfap, Gad1, Cnpase*) using SYBR Green PCR Master Mix (Applied Biosystems) were prepared, with the cDNA being evenly divided into each reaction. Percent de-enrichment was calculated by comparing relative *Ct* values between total and TRAP RNA samples for each set of primer. To assay LTP induction, 20 ng of total RNA and TRAP RNA for both basal and LTP conditions were reverse transcribed. Normalization of basal/LTP *Ct* values with HPRT1 was done before comparing fold-change of activity-dependent transcripts (*Arc, c-Fos*) through relative *Ct* values. To validate sequencing results, we collected a separate biological sample for both 60 and 120 min time points and performed RT-qPCR for the indicated transcripts. Some primer sequences were obtained from PrimerBank (http://pga.mgh.harvard.edu/primerbank) while others were designed to span exon-exon junctions.

### RNA sequencing library preparation and sequencing

~150 ng of TRAP RNA and total RNA were used in the library preparation, with a total of 12 samples per time point (biological triplicates for total basal/LTP and TRAP basal/LTP). RNA libraries were made using Illumina TruSeq RNA library preparation v2 kit. rRNA was first depleted from all samples using the Ribo-Zero Gold kit. Libraries were prepared according to manufacturer's instructions, skipping the oligodT purification step. Single-end (30 min time point) and paired-end sequencing (60 and 120 min time points) were performed using the Illumina Hiseq2500 system. Both library preparation and sequencing were done at the UCLA Neuroscience Genomics Core. Read lengths were 64 bp for single-end reads and 69 bp for paired-end reads, with an average of 45 million mapped reads per sample.

### RNA-seq, time-series, go enrichment analysis

RNA sequencing reads were mapped to the mm10 annotation of the mouse genome using STAR aligner (Dobin et al., 2013) and differential expression analysis was done with edgeR (Robinson et al., 2009). Reads were normalized by the trimmed-mean method for each time point before differential expression analysis. Significant differential expression used a cutoff of FDR < 0.1 and log_2_ fold-change of at least ±0.4. Hypergeometric tests were used to determine significance of overlap between DE transcripts of different variables.

Heatmaps were generated with gplots in R displaying all significant transcripts across all time points. The bottom 5% expressed transcripts (i.e., lowly expressed significant transcripts) were excluded as to minimize visual artifacts.

KEGG pathway analysis of up and downregulated transcripts was done using WebGestalt with a whole genome background list. All functional category enrichment analyses considered an adjusted *p*-value < 0.05 after BH correction to be significant, with a minimum of 4 genes per category. Up and downregulated transcripts from all time points were included.

Time series analysis was done with Short Time-series Expression Miner (STEM) (http://www.cs.cmu.edu/~jernst/stem/) using the fold-changes for transcripts that were significant in at least one time point. Gene ontology analysis was done using WebGestalt (http://www.webgestalt.org/option.php) on the temporal profiles derived from STEM analysis. All transcripts that had at least an average read number of 20 normalized reads by TRAP-seq across all time points was used as the background transcript list. For enrichment of TRAP-seq, RNA-seq, or both TRAP-seq and RNA-seq DE transcript function, PANTHER (http://pantherdb.org/) was also used to identify enriched categories and the genes associated with them with a background list derived from RNA-seq expression levels of 20 normalized reads on average across all samples and time points.

### UTR length and RBP motif enrichment analysis

Annotated 5′ and 3′ UTR lengths were obtained from BioMart using the Ensembl GRCm38 build. In cases of multiple annotated UTRs for a given gene, the average was taken and used for resampling. The annotated UTRs of brain-expressed transcripts from Kang et al. (2011) were compared against downregulated and upregulated transcripts using a Wilcox ranked test. This was also completed with a mouse-specific brain-expressed transcript list (Polymenidou et al., 2011) that yielded similar results but is not shown here. A large majority of transcripts overlapped between the two brain-expressed lists.

RBP motif enrichment analysis was completed with a resampling approach using 3′ UTR sequences of brain expressed transcripts because RBP motifs were underrepresented in simple scrambling of UTR sequences. A total of 244 mouse RNA binding protein motifs (taken from Ray et al., 2013) were tested on the 3′ UTR of upregulated and downregulated transcript sets, against a background of brain-expressed transcripts. We identified the numbers of RBP binding sites for each RBP within all 3′ UTRs of our up and downregulated genes, and generated a distribution of chance RBP motif numbers by resampling 3′ UTRs from the brain-expressed list 5,000 times and 1,000 times for the 5′ UTR list. We then compared the observed value against the distribution of expected numbers of RBP motif occurrences to generate a *p*-value. UTR length was accounted for during the resampling by fixing the total combined nucleotide length of all sampled UTRs to equal the total nucleotide length of all UTRs in up or downregulated transcript sets, as length variability had a measurable effect on the results. Significance criteria was *p* < 0.05 and a fold-enrichment > ± 0.2. An important note is that 5′ UTR lengths were much shorter than 3′ UTR lengths, leading to fewer overall observed binding sites for this permutation analysis.

### Cell-type specific enrichment analysis

We used the RNA-seq dataset from Zhang et al. (2014) to calculate cell type-enriched transcripts. It is important to note that the Zhang et al. study was done in P7 mice, while our study was done in 10–12 week old mice. Using FPKM numbers for neurons, myelinating oligodendrocytes, astrocytes, microglia, and endothelial cells, we calculated a neuronal enrichment factor by comparing FPKM for a given cell type against all other cell types that are expressed in the hippocampus. For example, for astrocytes we used a formula of (astrocyte FPKM) / (neuron + oligodendrocyte + microglia + endothelial cell FPKM) as a conservative way to determine which transcripts were astrocyte-enriched. We used an enrichment factor cutoff of 1.5 for all cell types. Transcripts we considered to be significantly DE in astrocytes had to be (1) astrocyte-enriched and (2) differentially expressed by a cutoff of FDR < 0.1 and a log2 fold-change of at least ± 0.4.

To determine neuron-enriched genes using TRAP-seq/RNA-seq data, we took the average of normalized basal read counts across all samples from all time points for each gene and found the ratio of TRAP-seq reads over RNA-seq reads. We considered a ratio of 1.5 to be neuron-enriched; this was empirically a stringent cutoff for neuron-enrichment because many known neuron-specific genes such as MAP2 and Arc were excluded from our neuron-enriched list. This was done to avoid including transcripts that may be expressed at significant levels in other cell types. From this list, we included a 10-read minimum cutoff to ensure genes with inflated enrichment ratios were excluded. Significant DE transcripts by TRAP-seq were then identified from this list and included in the analysis. If a transcript was DE at multiple time points (as was the case for many transcripts DE by both TRAP-seq/RNA-seq), we took the average fold-changes between all time points the transcript was DE. Significance testing of the log_2_FC differences between TRAP-seq and RNA-seq was calculated using a Wilcoxon signed rank test.

### Transcription-factor binding analysis

A list of enriched transcription factor targets and significantly enriched binding motifs was identified using oPOSSUM 3.0 (http://opossum.cisreg.ca/oPOSSUM3/) and TRANSFAC on BioBase, respectively. Significant transcripts identified through total RNA sequencing at any time point were included in the analysis, with a background list of >20 average reads transcripts at 120′ for oPOSSUM and >10 average reads for TRANSFAC. Graphical representation is for oPOSSUM results only, while results from both analyses are included in Tables S9A,B.

## Ethics statement

Use of mice in this study followed the recommendations of and protocol approved by the UCLA Institutional Animal Care and Use Committee.

## Author contributions

PC and KM designed experiments; TO and JA prepared hippocampal mini-slices; PC bred and characterized Ribotag-CamKIIα mice, performed cLTP stimulations, prepared samples and performed immunohistochemistry and qPCR; PC, RK, CB, and GC performed bioinformatics analysis; PC and KM wrote manuscript, with edits from TO, RK, and GC.

### Conflict of interest statement

The authors declare that the research was conducted in the absence of any commercial or financial relationships that could be construed as a potential conflict of interest.
