# Supplementary figures and images for: Mapping Gene Expression in Excitatory Neurons during Hippocampal Late-Phase Long-Term Potentiation
Source: Front Mol Neurosci. 2017 Feb 22;10:39. doi: 10.3389/fnmol.2017.00039 (PMC5319997; doi:10.3389/fnmol.2017.00039)

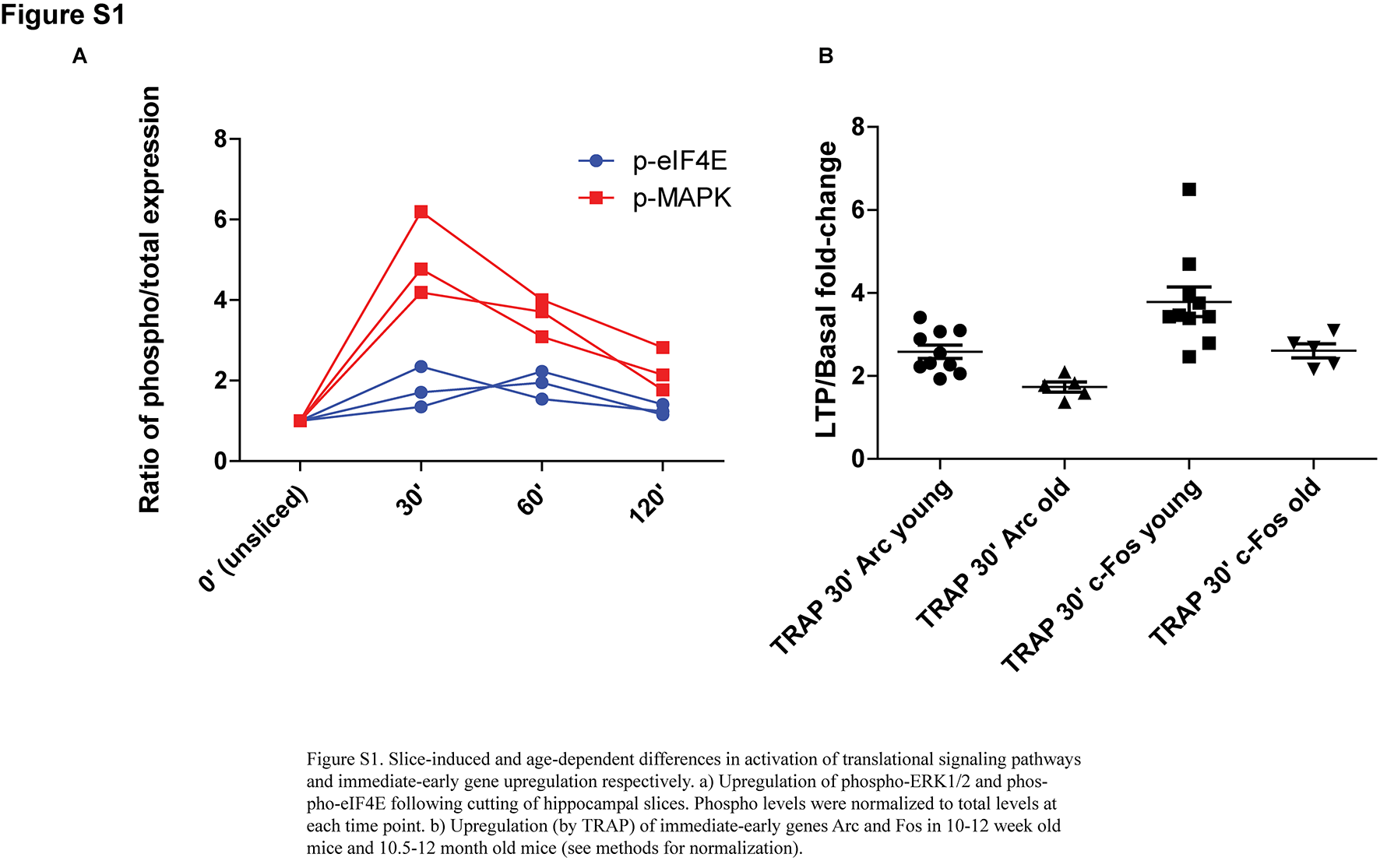

Supplement: Supplementary file 2 [file Image1.TIF]

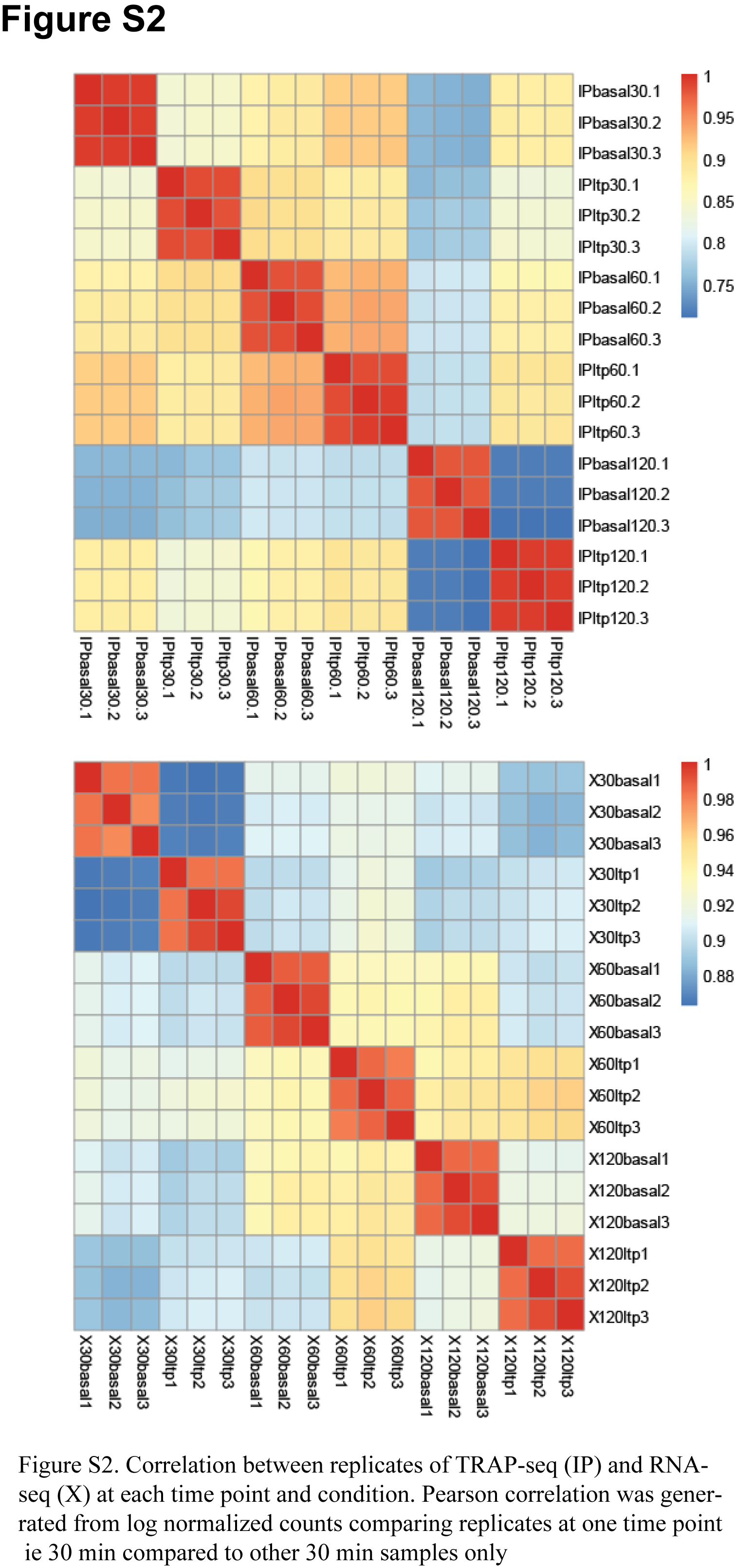

Supplement: Supplementary file 3 [file Image2.TIF]

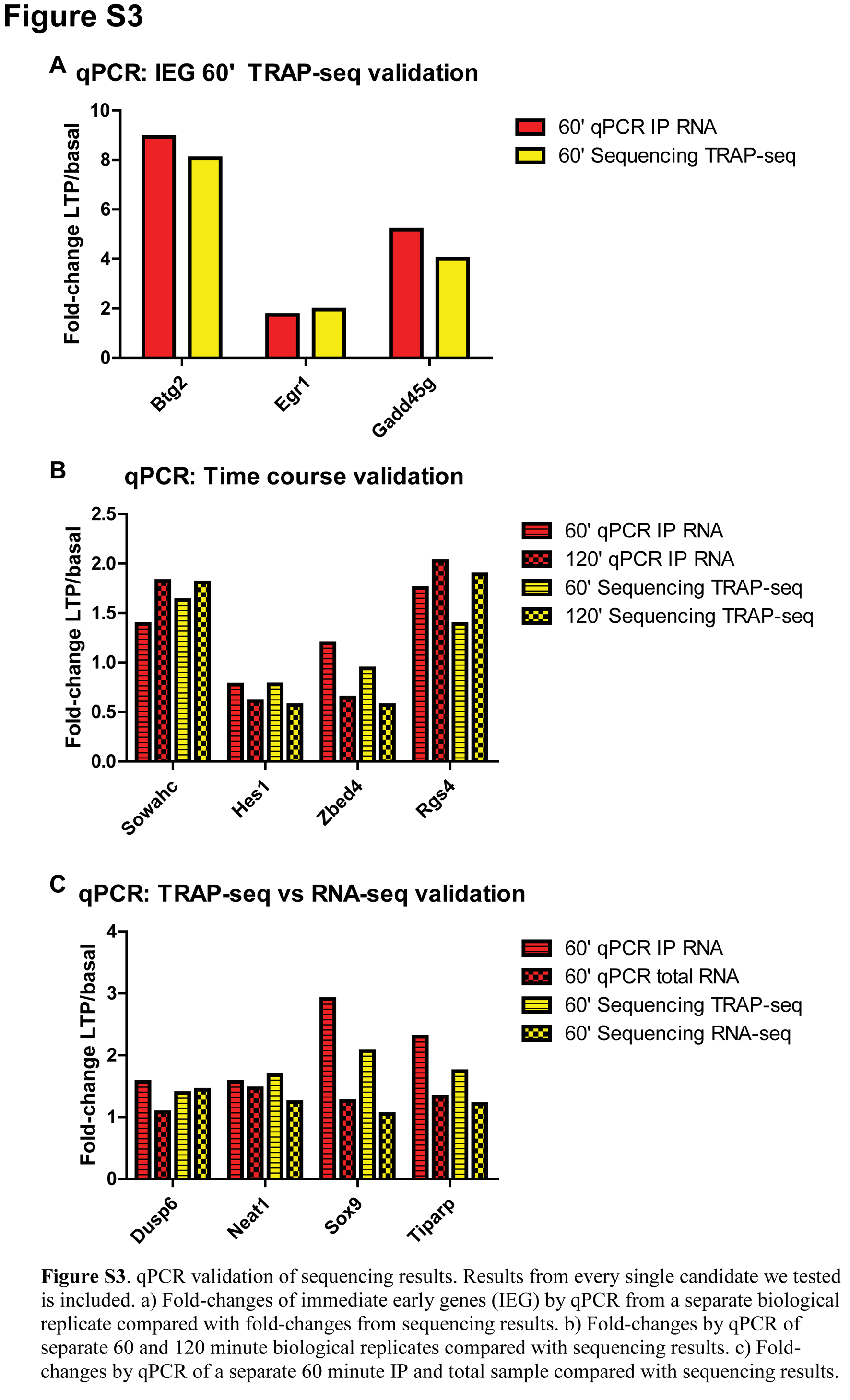

Supplement: Supplementary file 4 [file Image3.TIF]

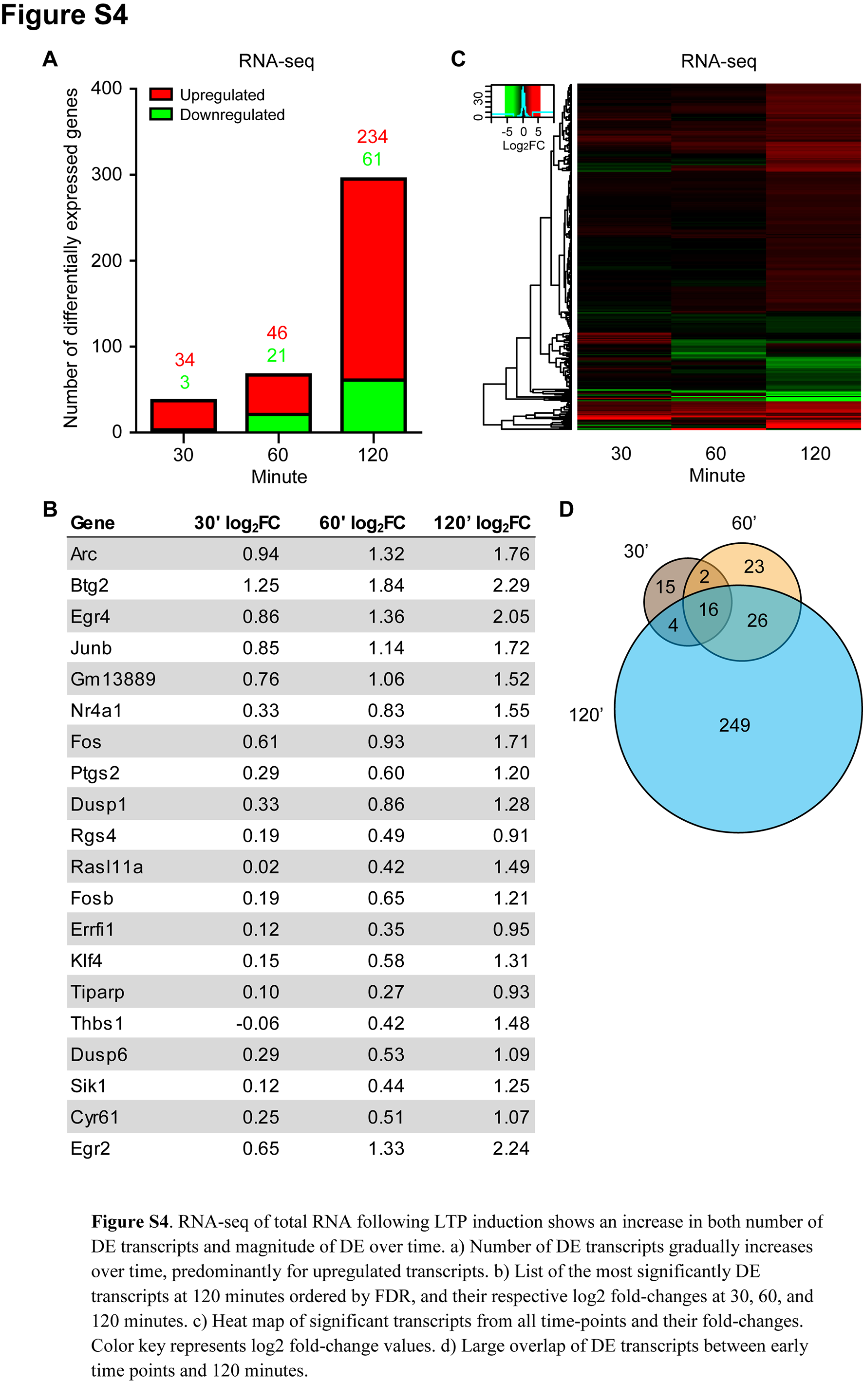

Supplement: Supplementary file 5 [file Image4.TIF]

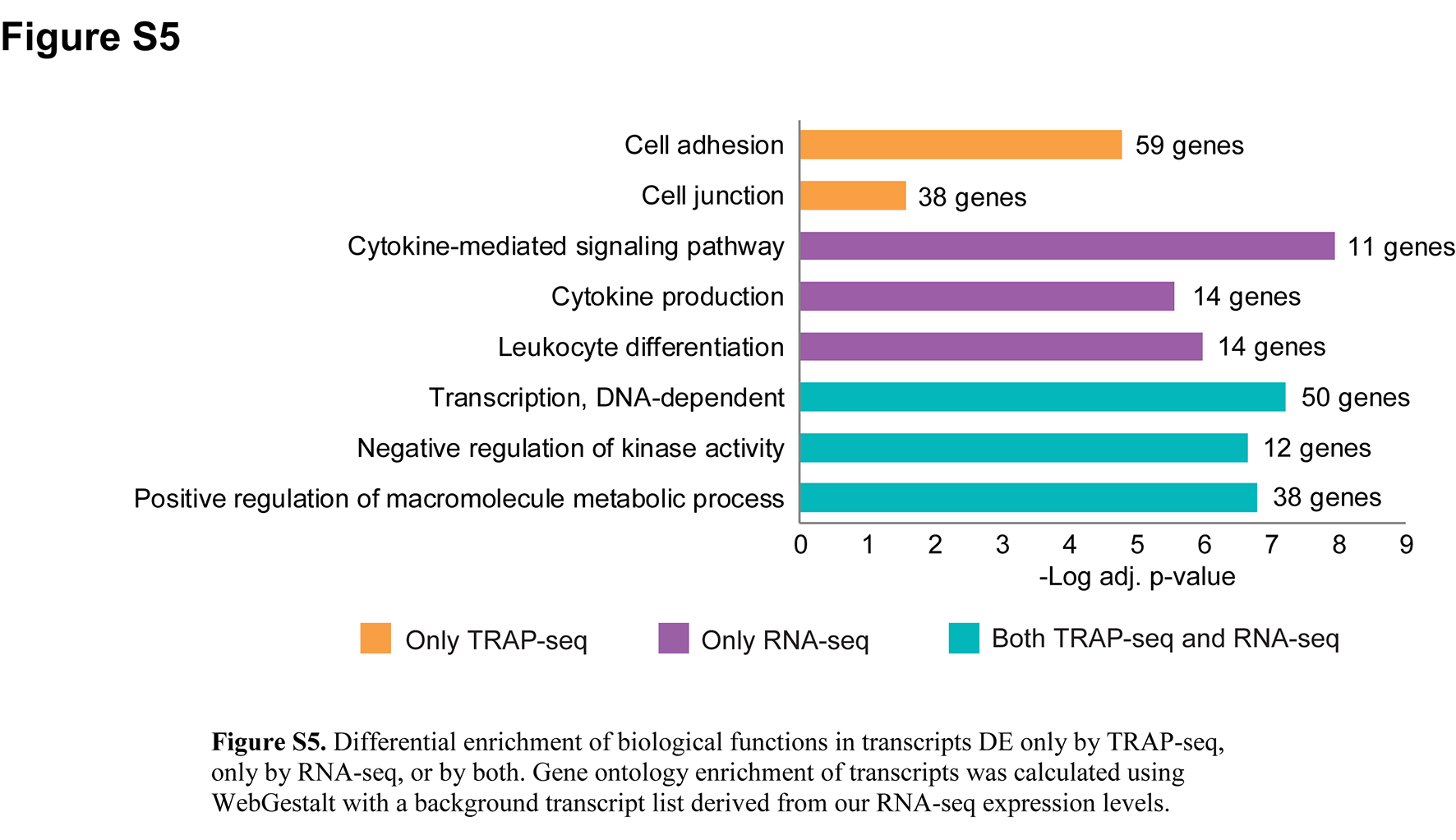

Supplement: Supplementary file 6 [file Image5.TIF]
